# Role of diffusion-weighted MRI in differentiation between benign and malignant anterior mediastinal masses

**DOI:** 10.3389/fonc.2022.985735

**Published:** 2022-10-13

**Authors:** Tran Thi Mai Thuy, Nguyen Truong Hoang Trang, Tran Thanh Vy, Vo Tan Duc, Nguyen Hoang Nam, Phan Cong Chien, Le Huu Hanh Nhi, Le Huu Nhat Minh

**Affiliations:** ^1^ Department of Diagnostic Imaging, University Medical Center, Ho Chi Minh City, Vietnam; ^2^ Department of Radiology, University of Medicine and Pharmacy at Ho Chi Minh City, Ho Chi Minh City, Vietnam; ^3^ Thoracic and Vascular Department, University Medical Center, Ho Chi Minh City, Vietnam; ^4^ Department of Thoracic and Cardiovascular Surgery, Faculty of Medicine, University of Medicine and Pharmacy at Ho Chi Minh City, Ho Chi Minh City, Vietnam; ^5^ Faculty of Medicine, University of Medicine and Pharmacy at Ho Chi Minh City, Ho Chi Minh City, Vietnam; ^6^ International Master/Ph.D. Program in Medicine, College of Medicine, Taipei Medical University, Taipei, Taiwan; ^7^ Global Clinical Scholars Research Training Program (GCSRT), Harvard Medical School, Boston, MA, United States; ^8^ Emergency Department, University Medical Center, Ho Chi Minh City, Vietnam

**Keywords:** mediastinal neoplasms, diffusion magnetic resonance imaging, thymic epithelial tumor, thymoma, lymphoma

## Abstract

Diffusion-weighted imaging (DWI) is considered to be a useful biomarker to characterize the cellularity of lesions, yet its application in the thorax to evaluate anterior mediastinal lesions has not been well investigated. The aims of our study were to describe the magnetic resonance (MR) characteristics of anterior mediastinal masses and to assess the role of apparent diffusion coefficient (ADC) value in distinguishing benign from malignant lesions of the anterior mediastinum. We conducted a retrospective cross-sectional study including 55 patients with anterior mediastinal masses who underwent preinterventional MR scanning with the following sequences: T1 VIBE DIXON pre and post-contrast, T2 HASTE, T2 TIRM, DWI-ADC map (b values of 0 and 2000 sec/mm^2^). The ADC measurements were obtained by two approaches: hot-spot ROI and whole-tumor histogram analysis. The lesions were grouped by three distinct ways: benign versus malignant, group A (benign lesions and type A, AB, B1 thymoma) versus group B (type B2, B3 thymoma and other malignant lesions), lymphoma versus other malignancies. The study was composed of 55 patients, with 5 benign lesions and 50 malignant lesions. The ADC_mean_, ADC_median_, ADC_10_, ADC_90_ in the histogram-based approach and the hot-spot-ROI-based mean ADC of the malignant lesions were significantly lower than those of benign lesions (P values< 0.05). The hot-spot-ROI-based mean ADC had the highest value in differentiation between benign and malignant mediastinal lesions, as well as between group A and group B; the ADC cutoffs (with sensitivity, specificity) to differentiate malignant from benign lesions and group A from group B were 1.17 x 10^-3^ mm^2^/sec (80%, 80%) and 0.99 x 10^-3^ mm^2^/sec (78.4%, 88.9%), respectively. The ADC values obtained by using the hot-spot-ROI-based and the histogram-based approaches are helpful in differentiating benign and malignant anterior mediastinal masses.

## Introduction

Despite being relatively rare entities, mediastinal masses present a diverse array of histopathology, ranging from benign lesions to malignancies. Anatomically, the anterior mediastinum contains the thymus, lymph nodes, fat tissue, nerves, blood vessels, and sometimes the thyroid gland descending from the neck. Masses in the anterior compartment of the mediastinum often originate from these structures (1).

Imaging plays a pivotal role in establishing the initial diagnosis and guiding the selection of supplementary tests needed to reach the final diagnosis. Because of superior soft tissue contrast, MRI is considered an ideal tool for evaluating mediastinal masses. Preoperative assessment of the involvement of the lesion with the pericardium, heart, spinal cord, and blood vessels is a common indication. In addition, non-contrast MRI is an alternative to evaluate mediastinal masses in patients who have contraindications to intravenous contrast. Chemical shift imaging has been shown to be useful in differentiating normal thymus and thymic hyperplasia from thymic neoplasms and lymphoma. Diffusion MRI is a novel technique that is able to assess physiologic differences between tissues based on the random motion of water protons (2). In diffusion-weighted imaging (DWI), the motion of water protons causes scattering of spins, which results in signal loss. The signal loss can be quantified by calculating the ADC, which reflects the specific diffusion capacity of biological tissue. The presence of diffusion obstacles, such as cell membranes, tight junctions, macromolecules, and cell organelles, will lead to decreased ADC. It is hypothesized that hypercellular tumors have low ADC values, whereas tissues with lower cell density (necrotic tissue, non-neoplastic tissue, etc.) have higher ADC values. Therefore, ADC values ​​can be used to distinguish necrotic tissue and benign lesions from malignancies. The cellular structure of a tumor is considered an indicator of its progression and response to treatment. In malignant lesions, the extracellular space is markedly narrowed due to increased cellularity, cell volume, and impaired cell membranes. Rapidly growing tumors have an increased quantity of organelles and cytoskeleton. The increase in cellular density and microstructures will in turn restrict the movement of water molecules (3, 4).

Classically, computed tomography is the imaging modality of choice to preoperatively evaluate prevascular mediastinal lesions. The knowledge regarding MRI features of these masses is still considered to be modest because of their paucity. There have been published studies on the role of MRI in evaluating anterior mediastinal masses, some of which stressed the value of DWI in differentiating benign lesions and malignancies (5, 6). Yet only a few studies tried various approaches to obtain the ADCs or to group the lesions. The present study was carried out with the aims of characterizing anterior mediastinal masses on MRI and investigating the role of ADCs in differentiating benign and malignant anterior mediastinal lesions, which were obtained through two distinct manners: the hot-spot-ROI and the whole-tumor histogram analysis.

## Materials and methods

**Figure 1:**
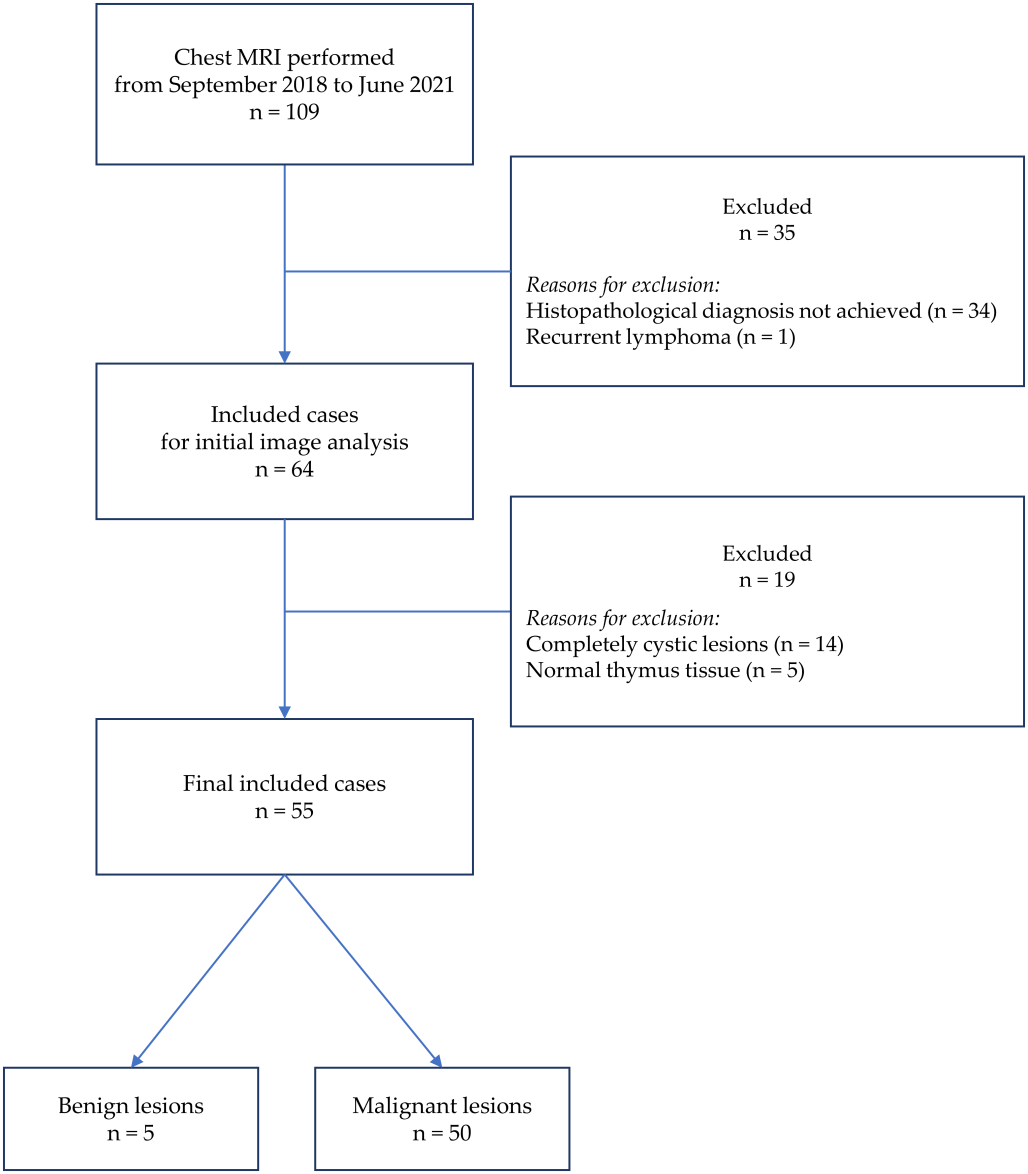
The flow diagram of sample selecting.

This is a retrospective cross-sectional study including 55 patients with anterior mediastinal masses who underwent preinterventional chest MRI at an institution from September 2018 to June 2021. The histopathological diagnoses were achieved with samples taken from operations or biopsies. The exclusion criteria were (a) lesions without solid components (completely cystic lesions) and (b) recurrent cases. Patient selection is demonstrated in **Figure 1**. We thoroughly searched the database of our institution and found that 109 chest MRIs were conducted in the mentioned period to investigate anterior mediastinal lesions. Among 109 patients, we first excluded 35 cases, including 34 cases in which the final histopathology was not achieved (due to the lack of operation or biopsy) and 1 case of recurrent lymphoma. After carefully reviewing the images of the remaining 74 cases, we continued excluding 19 cases, including 14 completely cystic lesions and 5 cases in which the histopathological diagnoses were “thymic remnant”. These 5 “thymic remnant” patients had thymectomy to treat their myasthenia gravis; the 14 completely cystic lesions were 6 mature teratomas, 5 congenital benign cysts, and 3 thymomas.

All studies were performed on a 3 T MR unit (Magnetom, Siemens, Germany) at the University Medical Center, Ho Chi Minh City. The MRI protocol for each patient included T2 HASTE, T2 HASTIRM, DWI, T1 VIBE DIXON before and after gadoterate meglumine injection, with the following parameters. Axial T2 HASTE: TR = 1000 msec, TE = 92 msec, slice thickness: 6 mm, field of view: 260x360, and matrix: 240x320. Sagittal T2 HASTE: TR = 600 msec, TE = 27 msec, slice thickness: 8 mm, field of view: 350x350, and matrix: 256x320. Coronal T2 HASTE: TR = 600 msec, TE = 26 msec, slice thickness: 8 mm, field of view: 400x400, and matrix: 256x320. Axial T2 HASTIRM: TR = 1600 msec, TE = 86 msec, slice thickness: 6.5 mm, field of view: 300x380, and matrix: 260x320. Axial T1 opposed-phase: TR = 4.2 msec, TE = 1.3 msec, slice thickness: 3 mm, field of view: 280x380, and matrix: 240x320. Axial T1 in-phase: TR = 4.2 msec, TE = 2.6 msec, slice thickness: 3 mm, field of view: 280x380, and matrix: 240x320. Axial DWI: TR = 6500 msec, TE = 72 msec, slice thickness: 6 mm, field of view: 320x400, and matrix: 120x150. Diffusion-weighted images were obtained by using an echo-planar imaging sequence with b values of 0 and 2000 sec/mm^2^. ADC maps were constructed by the machine’s software and displayed simultaneously after DWI images were acquired.

MR images were examined on the Picture Archiving and Communications System (PACS) of the University Medical Center Ho Chi Minh City. The characteristics investigated included size, border, presence of necrotic or cystic component, and presence of intra-lesional fat. These characteristics are analyzed based on T1 DIXON images before and after gadolinium injection, T2 HASTE, T2 TIRM. To accurately quantify the presence of fat, we used the SII, which stands for Signal Intensity Index. Fat composition was determined by comparing the signal intensity of the lesion on in-phase and opposed-phase T1 DIXON images. The region of interest (ROI) was placed on the slice with the largest tumor diameter, with the ROI area occupying approximately three-fourths of the tumor. The SII was calculated by the following formula:


$$SII=\frac{SI_{in}-SI_{op}}{SI_{in}}\times 100$$

**Figure 2:**
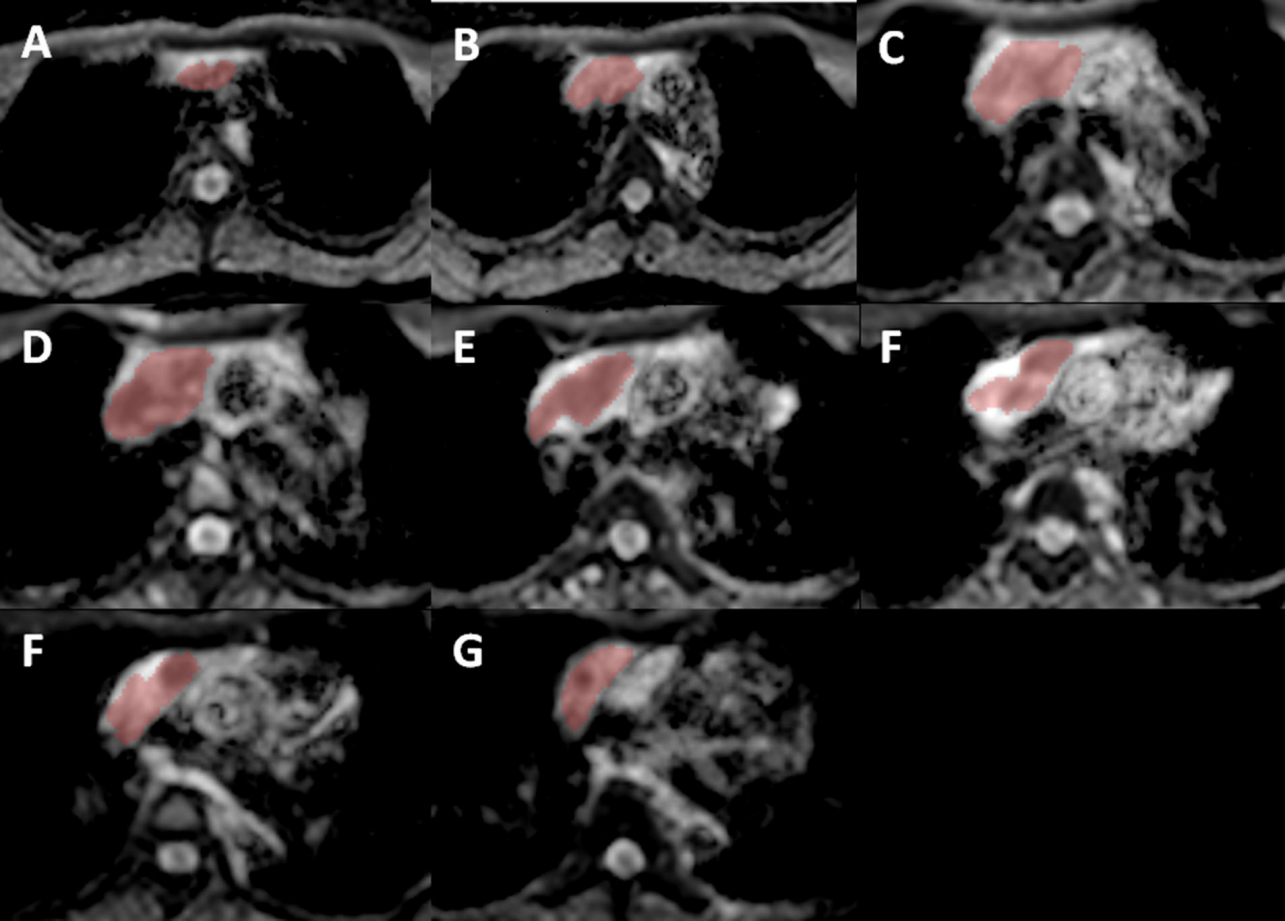
Using FireVoxel, ROIs were manually placed on consecutive slices of the ADC map across the whole lesion, from **(A)** being the most cephalic and **(G)** being the most caudal slice. Images from **(B–F)** are intermediate slices between these two following that direction/order.

**Figure 3:**
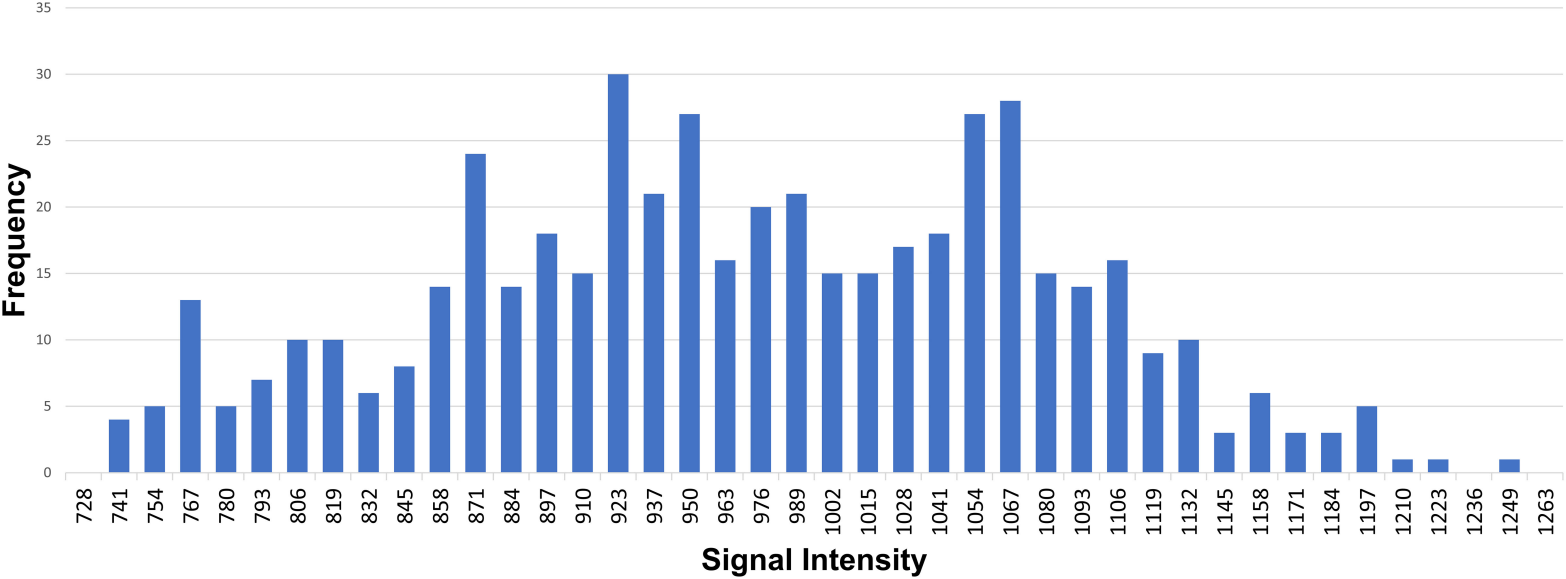
An example of whole-tumor histogram analysis of the ADC map. A 35-year-old female patient with primary mediastinal large B-cell lymphoma.

We implemented two approaches to obtain the ADC values: the hot-spot-ROI and the whole-tumor-histogram. For the hot-spot-ROI approach, three ROIs (area of 0.5 cm^2^) were manually placed on the ADC map at three different sites of solid tissue, which were the most hypointense on the ADC map and correspondingly hyperintense on the trace image (b value of 2000 sec/mm^2^). Areas of fat, hemorrhage, necrosis, and cystic components were excluded from the ROI. The average of three ADC values was then recorded. For the whole-tumor histogram analysis, we used FireVoxel, a non-commercial software developed by the Center for Biomedical Imaging, Department of Radiology, New York University. ROIs were manually drawn on each slice across the whole tumor (**Figure 2**). Large areas of fat, necrosis, cysts, and hemorrhage were removed from the ROI. The software then constructed the histogram analysis of ADC values; four measurements, including ADC_mean_, ADC_median_, ADC_10_ (the 10th percentile of ADC), and ADC_90_ (the 90th percentile of ADC), were recorded (**Figure 3**).

We grouped the lesions using three distinct methods. First, the lesions were categorized as benign or malignant according to the WHO classification of thoracic tumors published in 2021 (7). The lesions were additionally divided in two other ways: 1. group A (benign lesions and type A, AB, B1 thymoma) versus group B (type B2, B3 thymoma and other malignancies); 2. lymphoma versus other malignancies. Based on the proposal of Jeong et al. (8), we separated type A, AB, and B1 thymomas (low-risk thymomas) from type B2 and B3 thymomas (high-risk thymomas) in the concept of group A versus group B.

Data were shown as frequency and percentage for qualitative variables; for normally distributed quantitative variables, data were shown as mean values and standard deviation; for non-normally distributed quantitative variables, data were shown as median and interquartile range. We used the chi-squared test and Fisher’s exact test to compare proportions. The Mann-Whitney U test was used to compare non-normally distributed means. Receiver operating characteristic (ROC) curves were constructed to detect the cutoff ADC value with a calculation of sensitivity, specificity, and accuracy. The data were analyzed with STATA version 14.1 (STATA Corp., Texas, USA). P values of 0.05 or less indicated statistical significance.

## Result

Our study included 55 patients (23 males and 32 females) with 5 benign lesions (9.1%) and 50 malignant lesions (90.9%). The pathologies of benign lesions were thymic hyperplasia, mature teratoma, and Castleman disease. High-risk thymoma was the most common pathology of the malignant group, constituting 32%. Pathological diagnoses are presented in **Table 1**. The age of the benign group and the malignant group was 26.8 ± 10.5 (range 13–37) and 49.5 ± 16.4 (range 15–82), consecutively. The presence of myasthenia gravis was observed in 15 cases (27.3%, 15/55). Specifically, myasthenia gravis was present in 100% (2/2) cases of thymic hyperplasia, 38.5% (5/13) cases of low-risk thymoma, and 50% (8/16) cases of high-risk thymoma.

**Table 1:** Pathological diagnoses.

Pathology	Quantity	Percentage
***Benign lesions***	***5***	
Thymic hyperplasia	2	40%
Mature teratoma	2	40%
Castleman disease	1	20%
***Malignant lesions***	***50***	
Low-risk thymoma	13	26%
High-risk thymoma	16	32%
Thymic carcinoma	8	16%
Thymic NET	2	4%
Malignant GCT	4	8%
Lymphoma	7	14%

NET, Neuroendocrine tumor; GCT, Germ cell tumor.Bold and italics values indicated the values of the two main groups (benign lesions and malignant lesions).

Out of benign lesions, teratoma had the largest diameter of 114.5 mm, while Castleman disease had the smallest diameter of 36 mm. Out of malignant lesions, malignant germ cell tumor had the largest diameter of 124.8 mm, low-risk thymoma had the smallest diameter of 54.2 mm. Thymic hyperplasia and Castleman disease had regular margins and no cystic component. All GCTs (mature teratomas and malignant germ cell tumors) had cystic components and lobulated margins. Among thymic epithelial tumors (low-risk thymoma, high-risk thymoma, and thymic carcinoma), rate of lesions containing fluid and having a lobulated margin increased correspondingly to the degree of malignancy. Most lymphoma cases did not contain cystic components and had quite equal rates of lobulated and regular margins. Thymic hyperplasia and teratoma had high SII, 60.5% and 41.7%, respectively. Other tumors have low SII, ranging from -5.1 to 7.4. **Table 2** summarizes the detailed relationship between MRI findings and pathological diagnostics.

**Table 2:** Relationship between MRI findings and pathological diagnostics.

Pathology	Diameter ^1^ (mm)	Margin^2^		Cystic^2^		SII^1^
		Regular	Lobulated	No	Yes	
***Benign (n = 5)***	***73.4 ± 39.3***	***3*** *(60)*	***2 (40)***	***3 (60)***	***2 (40)***	***39.9 ± 20.2***
Thymic hyperplasia	49.5 ± 7.8	2 (100)	0 (0)	2 (100)	0 (0)	60.5 ± 44.2
Mature teratoma	114.5 ± 20.5	0 (0)	2 (100)	0 (0)	2 (100)	41.7 ± 6.4
Castleman disease	39	1 (100)	0 (0)	1 (100)	0 (0)	-5.1
***Malignant (n = 50)***	***71.9 ± 32.3***	***20 (40)***	***30 (60)***	***30 (60)***	***20 (40)***	***2.1 ± 8.9***
Low-risk thymoma	54.2 ± 20.7	10 (76.9)	3 (23.1)	9 (69.2)	4 (30.8)	7.4 ± 17.8
High-risk thymoma	63.4 ± 31.7	6 (37.5)	10 (62.5)	11 (68.7)	5 (31.3)	-0.8 ± 6.5
Thymic carcinoma	74.6 ± 19.2	1 (12.5)	7 (87.5)	4 (50)	4 (50)	2.6 ± 5.3
Thymic NET	88.5 ± 24.7	0 (0)	2 (100)	0 (0)	2 (100)	-4.3 ± 9.1
Malignant GCT	124.8 ± 35.1	0 (0)	4(100)	0 (0)	4 (100)	1.4 ± 3.9
Lymphoma	86.1 ± 30.7	3 (42.9)	4 (57.1)	6 (85.7)	1 (14.3)	0.5 ± 4.7

**^1^** Data are reported as mean ± standard deviation.

**^2^** Data are reported as frequency (percentage).

SII, Signal intensity index; NET, Neuroendocrine tumor; GCT, germ cell tumor. Bold and italics values indicated the values of the two main groups (benign lesions and malignant lesions).

The differences in hot-spot-ROI-based mean ADC between benign lesions and malignancies, group A and B lesions, lymphoma and other malignant tumors were all statistically significant. Group B exhibited significantly lower ADC_10_, ADC_median_, ADC_mean_, and ADC_90_ (p values< 0.05) compared to group A. There were no significant differences in the ADC_10_, ADC_median_, ADC_mean_ and ADC_90_ between benign and malignant tumors, lymphoma and other malignant tumors (p values > 0.05). **Table 3** shows the ADC values of different benign and malignant mediastinal lesions obtained by two approaches: hot-spot ROI and whole-lesion histogram.

**Table 3:** Hot-spot-ROI-based and whole-tumor-histogram-based ADC measurements.

Pathology	Hot-spot-ROI-based mean ADC (x10^-3^ mm^2^/sec)	Histogram-based ADCs (x 10^-3^ mm^2^/sec)			
		ADC_mean_	ADC_median_	ADC_10_	ADC_90_
***Benign (n = 5)***	***1.46 ± 0.23***	***1.28 ± 0.09***	***1.20 ± 0.12***	***1.03 ± 0.11***	***1.54 ± 0.16***
Thymic hyperplasia	1.47 ± 0.34	1.33 ± 0.12	1.34 ± 0.16	1.08 ± 0.05	1.55 ± 0.17
Mature teratoma	1.46 ± 0.2	1.24 ± 0.01	1.23 ± 0.04	0.99 ± 0.16	1.52 ± 0.21
Castleman disease	0.8	0.84	0.82	0.7	1.05
***Malignant (n = 50)***	***0.96 ± 0.29***	***1.05 ± 0.23***	***1.05 ± 0.24***	***0.83 ± 0.2***	***1.28 ± 0.26***
Low-risk thymoma	1.19 ± 0.18	1.2 ± 0.2	1.2 ± 0.2	0.97 ± 0.2	1.45 ± 0.2
High-risk thymoma	0.98 ± 0.36	1.05 ± 0.28	1.06 ± 0.29	0.81 ± 0.24	1.3 ± 0.31
Thymic carcinoma	0.9 ± 0.17	0.93 ± 0.15	0.93 ± 0.15	0.74 ± 0.14	1.12 ± 0.17
Thymic NET	0.6 ± 0.04	0.83 ± 0.04	0.82 ± 0.04	0.63 ± 0.03	1.03 ± 0.03
Malignant GCT	0.8 ± 0.16	1.1 ± 0.17	1.08 ± 0.16	0.82 ± 0.09	1.33 ± 0.19
Lymphoma	0.75 ± 0.12	0.95 ± 0.16	0.94 ± 0.17	0.76 ± 0.13	1.16 ± 0.2

Data are reported as mean ± standard deviation.

ADC, Apparent diffusion coefficient; NET, Neuroendocrine tumor; GCT, Germ cell tumor. Bold and italics values indicated the values of the two main groups (benign lesions and malignant lesions).

ROC curves were constructed to determine the diagnostic value of hot-spot-ROI-based mean ADC and histogram-based ADC parameters for differentiating benign lesions and malignant lesions, group A and group B, lymphoma and other malignant tumors, with the calculation of the cutoff value, sensitivity, specificity, and area under the ROC curve (AUC). Results are presented in **Table 4**.

**Table 4:** Diagnostic performance of hot-spot-ROI-based and histogram-based ADC measurements for differentiating between benign and malignant lesions, group A and B, lymphoma and other malignant tumors.

Benign and malignant			AUC (95% CI)	Cutoff	Sensitivity	Specificity	Accuracy
**Hot-spot-ROI mean ADC**			0.794 (0.681–0.853)	1.17	80%	80%	80%
**Histogram-based ADC parameters**	**ADC_mean_**		0.760 (0.678–0.844)	1.22	78%	81%	70.1%
	**ADC_median_**		0.782 (0.670–0.852)	1.18	76%	81%	72.9%
	**ADC_10_**		0,790 (0.669–0.872)	0.85	75.7%	72.2%	74%
	**ADC_90_**		0,773 (0.673–0.850)	1.36	62.2%	82%	75%
***Group A and B***			**AUC (95% CI)**	**Cutoff**	**Sensitivity**	**Specificity**	**Accuracy**
**Hot-spot-ROI mean ADC**			0.843 (0.721–0.889)	0.99	78.4%	88.9%	81.8%
**Histogram-based ADC parameters**		**ADC_mean_**	0.755 (0.678–0.841)	1.04	64.9%	83.3%	70.9%
		**ADC_median_**	0.752 (0.675–0.867)	1.03	64.9%	83.3%	70.9%
		**ADC_10_**	0.758 (0.669–0.872)	0.85	75.7%	72.2%	74.6%
		**ADC_90_**	0.755 (0.672–0.882)	1.22	62.2%	88.9%	71%
***Lymphoma and other malignant tumors***			**AUC (95% CI)**	**Cutoff**	**Sensitivity**	**Specificity**	**Accuracy**
**Hot-spot-ROI mean ADC**			0.772 (0.679–0.845)	0.91	100%	60.5%	66%
**Histogram-based ADC parameters**	**ADC_mean_**		0.762 (0.677–0.830)	0.99	95.1%	60.2%	65.4%
	**ADC_median_**		0.763 (0.672–0.839)	0.98	95.2%	60.1%	65.5%
	**ADC_10_**		0.771 (0.669–0.872)	0.88	99.8%	60.4%	65%
	**ADC_90_**		0.750 (0.672–0.882)	1.01	95.0%	59.5%	64.9%

CI, confidence interval.

All cutoffs were selected as the points achieving the maximal Youden index. Bold and italics values indicated the values of the two main groups (benign lesions and malignant lesions).

**Figure 4:**
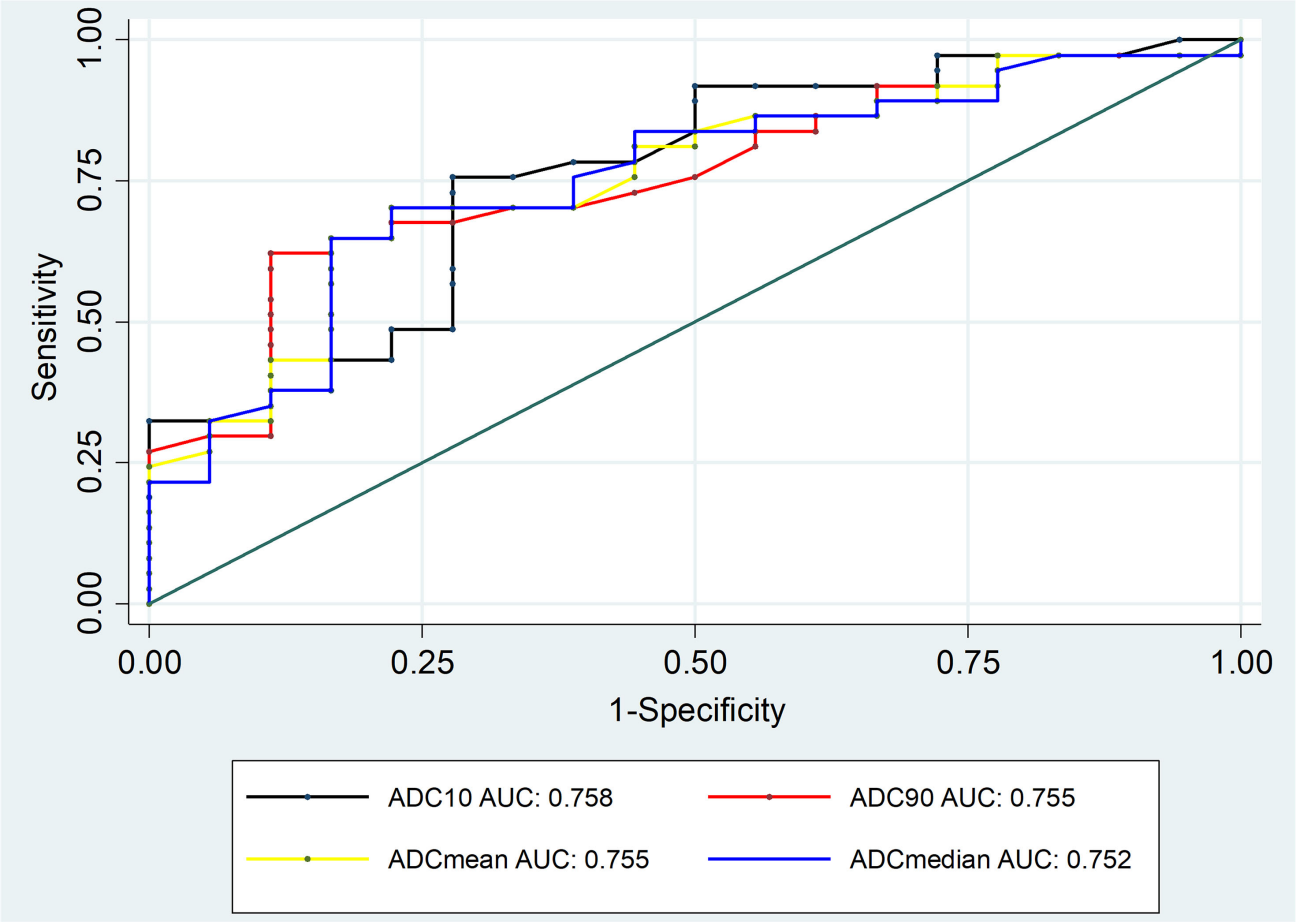
ROC curves of histogram-based ADC measurements for differentiating between group A and group B lesions.

Regarding differentiating between group A and B lesions, the mean ADC value based on hot-spot-ROI with the cutoff value of 1.17 x 10^-3^ mm^2^/sec had the highest sensitivity, specificity, and accuracy. Out of the ADC parameters obtained from whole-tumor histogram analysis, the ADC_10_ achieved the highest AUC, followed by ADC_mean_, ADC_90_ and ADC_median_ (**Figures 3**, **4**).

## Discussion

The largest diameter, contour and presence of cystic components of anterior mediastinal tumors in our study were similar to their macroscopic appearance and radiographic features described in previous studies and textbooks (9–11).

**Figure 5:**
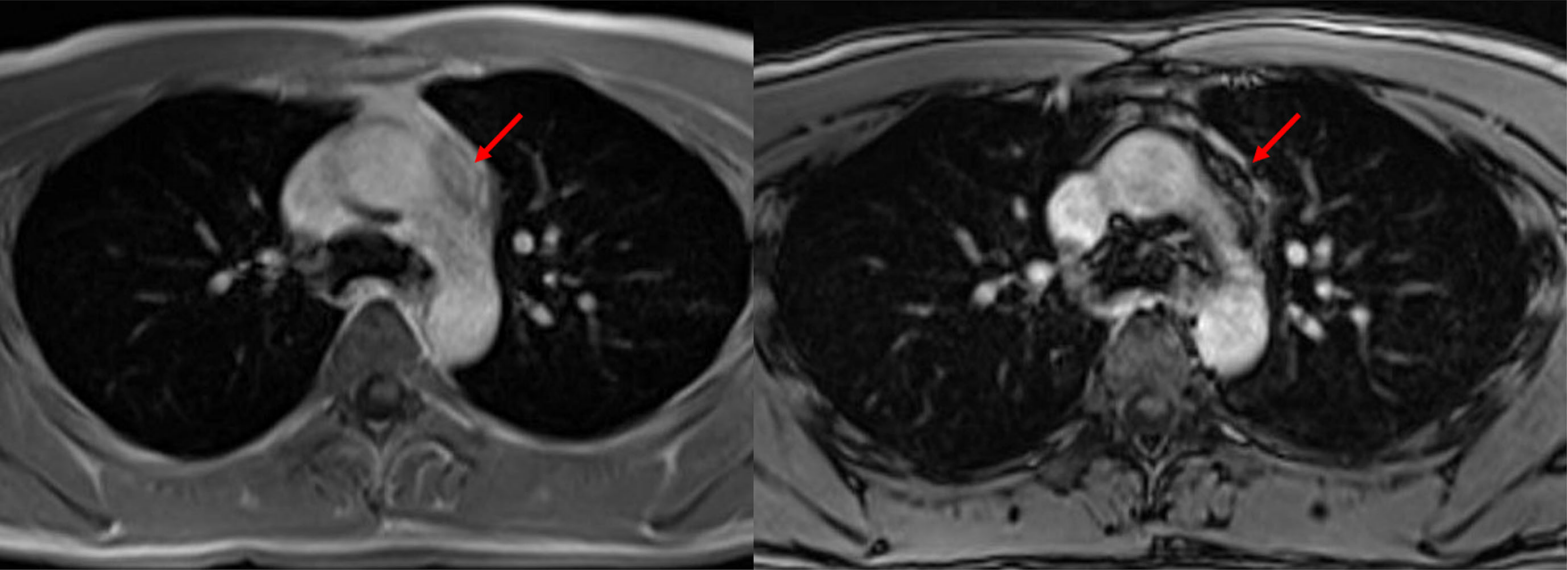
Sclerosing thymoma. A 27-year-old male patient with sclerosing thymoma (arrow). Placing a ROI in the same area on in-phase (left) and opposed-phase (right) T1-weighted images to identify SII, which was 64.6%.

Our study showed that the average SII of thymic hyperplasia was 60.5%, and this result was close to the result of M. Priola et al, in which the average SII of thymic hyperplasia was 45.9% (12). The mean SII value of mature teratoma was 41.7%, which was correlated well with the fat component in these lesions. Classically, Castleman disease and malignant tumors in the anterior mediastinum do not have any macroscopic or microscopic fat components; the low average SIIs of these lesions in our study correlated well with that fact. The low SII of malignant tumors in our study was similar to the result of M. Priola et al. (12). M. Priola et al. reported that SII could perfectly distinguish malignant tumors from thymic hyperplasia using a cutoff point of 8.92% (sensitivity and specificity 100%), and no overlap was found for SII between groups (12). However, in our study, one case pathologically diagnosed as sclerosing thymoma had a high SII value, which was overlapped with SII values in teratoma and thymic hyperplasia groups (**Figures 4**, **5**). It can be explained that when placing a ROI within the tumor to determine SII, we accidentally chose the area having both tumoral tissue and normal thymic tissue, so that fat in normal thymic tissue raised the SII value. In a case report of a sclerosing thymoma by Li Xin et al, the surgical specimen contained adipose tissue, incompletely involuted thymic tissue and tumor components (13). If the SII value of sclerosing thymoma was the outlier, there would be no overlapped values between teratoma or thymic hyperplasia group and other tumors group. Chemical shift MRI is a valuable tool for detecting and quantifying the fat component of mediastinal lesions.

**Figure 6:**
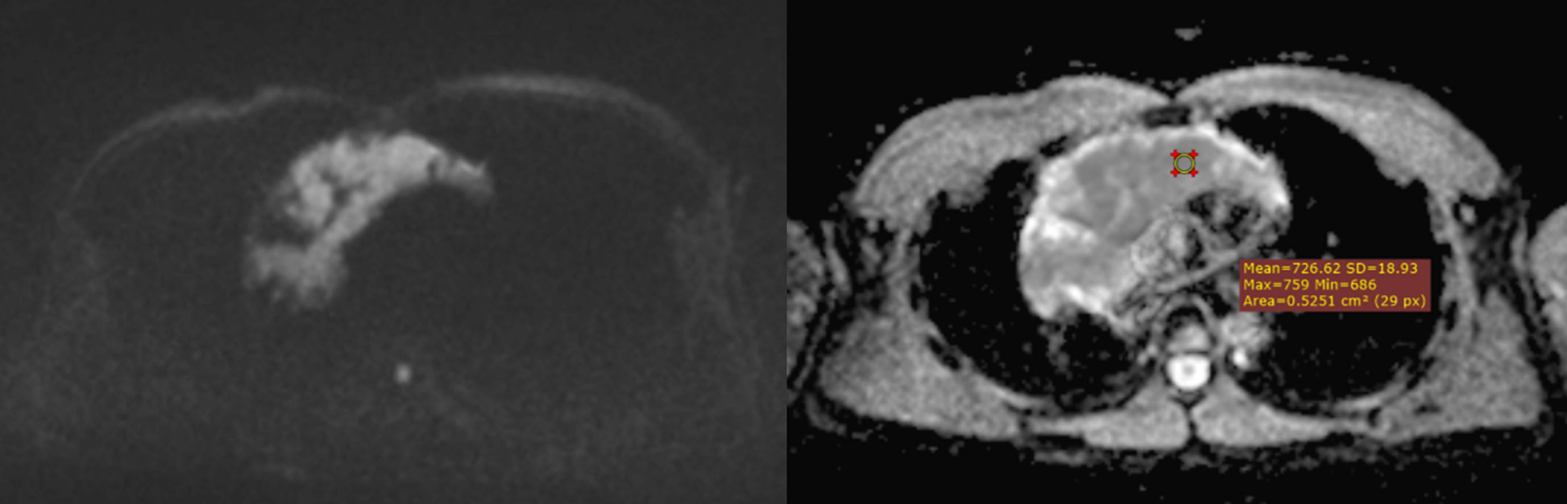
A mediastinal seminoma (malignant GCT) presurgically diagnosed as a malignant tumor based on its markedly low ADC value. The mean of 3 ADC values obtained by using the hot-spot-ROI-based approach was 0.7 x 10-3 mm2/sec.

Using the hot-spot-ROI-based approach, the cutoff of mean ADC to distinguish malignant from benign lesions was 1.17 x 10^-3^ mm^2^/sec, AUC was 0.794, sensitivity and specificity were both 80% (**Figures 5**, **6**). This result is concordant with that of Raafat et al. who reported that the cutoff ADC value for the differentiation between malignant and benign mediastinal lesions was 1.11 x 10^-3^ mm^2^/sec, with sensitivity of 90.9% and specificity of 100% (5). However, our result is discordant with the work of Usuda, who reported a higher cutoff value of 2.21 x 10^-3^ mm^2^/sec for discriminating between malignant and benign mediastinal tumors (6). The differences in sample size and ADC measurement method between our study and the work of Usuda may explain the dissimilarities between the two results. There were some completely cystic lesions in the study of Usuda (6), whereas our study excluded these. In addition, when measuring the ADC values, Usuda et al. placed ROIs around the margin of the tumors without excluding fatty, necrotic, cystic, and hemorrhagic areas, leading to the higher ADC values.

**Figure 7:**
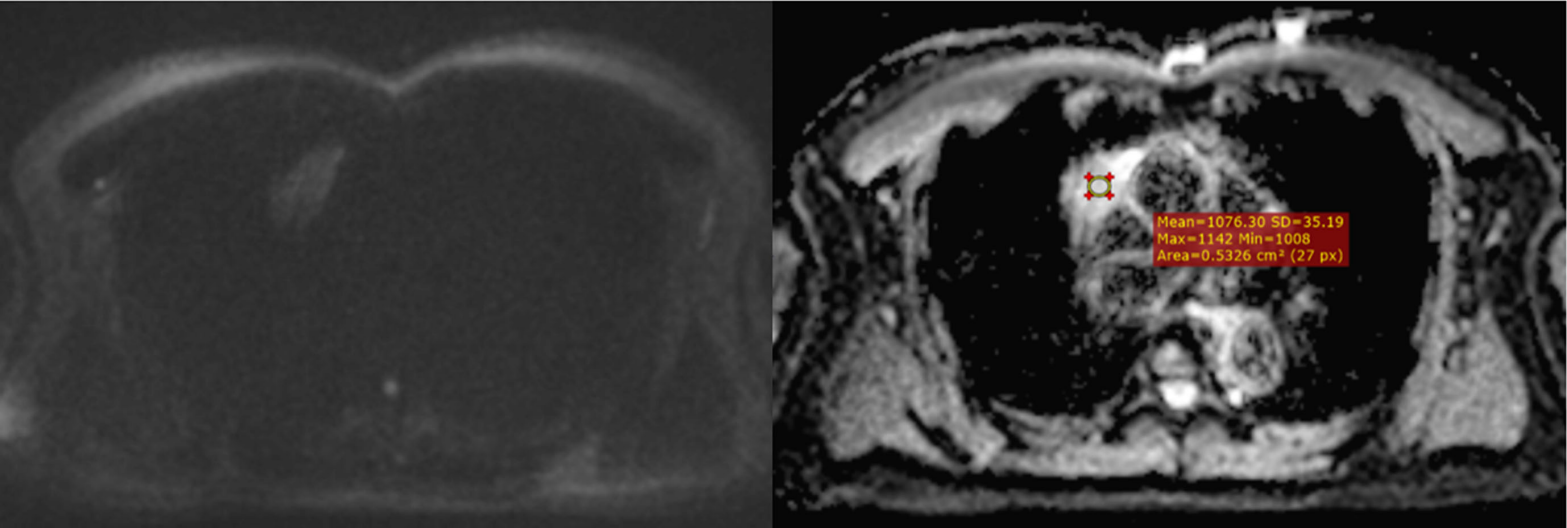
Type AB thymoma on DWI/ADC map. A case of confirmed type AB thymoma, correctly classified as a low-risk thymoma on pre-surgical MRI. The mean of 3 ADC values obtained by using the hot-spot-ROI-based approach was 1.05 x 10-3 mm2/sec.

**Figure 8:**
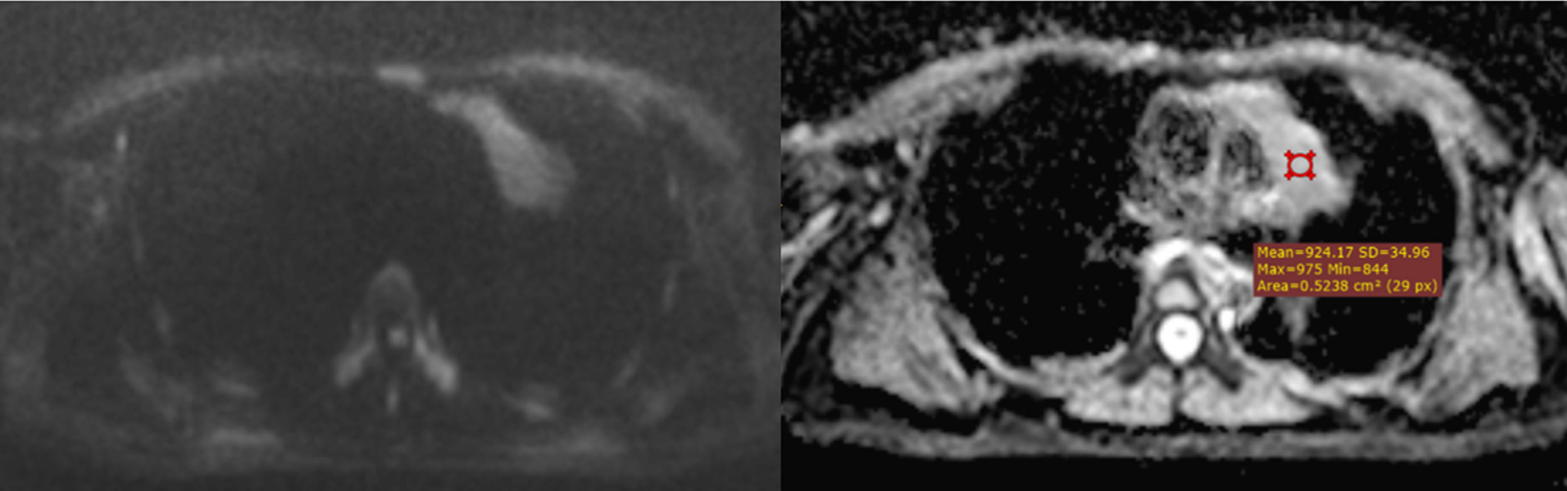
A type B3 thymoma, correctly predicted as a high-risk thymoma on DWI/ADC map. The mean of 3 ADC values obtained by using the hot-spot-ROI-based approach was 0.91 x 10-3 mm2/sec, which was lower than the cutoff of 0.99 x 10-3 mm2/sec.

**Figure 9:**
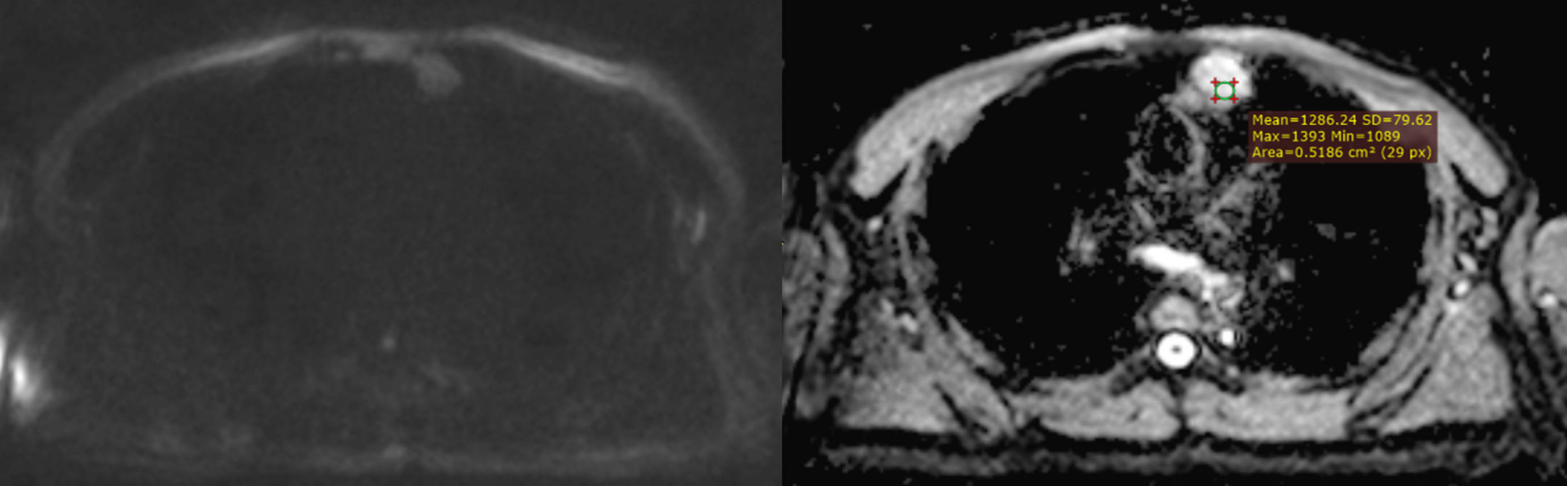
A case of false prediction using the hot-spot-ROI-based ADC cutoff of 0.99 x 10^-3^ mm^2^/sec. A pathologically-proven type B3 thymoma was falsely presumed as a low-risk thymoma according to its rather high ADC value. The mean of 3 ADC values obtained by using the hot-spot-ROI-based approach was 1.15 x 10-3 mm2/sec, which was obviously higher than the cutoff of 0.99 x 10-3 mm2/sec.

According to the WHO classification of thoracic tumors published in 2021 (7), almost all thymic epithelial tumors (TETs) are considered to have “aggressive behavior”. The two exceptions are lipofibroadenoma and micronodular thymoma with lymphoid stroma, yet these rare pathologies were not encountered in our study. Therefore, we put all thymomas into the category of “malignant.” The concept of dividing thymomas into low-risk (type A, AB and B1) and high-risk (type B2 and B3) was first introduced by Jeong et al. (8) thanks to its implications for prognosticating and treatment planning. Low-risk thymoma patients have more favorable prognosis than ones with high-risk thymoma (14), as well as low-risk thymomas are often detected at early Masaoka-Koga stages, leading to their lower rate of requiring adjuvant or neoadjuvant therapy. The classification was followed by several studies investigating the correlations between TET subtypes and their imaging features. Razek (15), Priola (16), and Shen (17) applied the division concept and established ADC cutoffs to distinguish low-risk thymoma from the remaining TETs. For these reasons, in the present study, we proposed another method of lesion categorization along with benign conditions versus malignancies: group A versus group B. The absolute quantity and rate of truly benign anterior mediastinal masses are modest, so the proposed classification may provide a different approach to anterior mediastinal lesions. When comparing the hot-spot-ROI-based mean ADC between the two groups, we found the mean ADC value of group B was significantly lower. With the cutoff of 0.99 x 10^-3^ mm^2^/sec, the distinction was achieved with a sensitivity of 78.4%, specificity of 88.9%, and accuracy of 81.8% (**Figures 6**–**9**). Having the relatively similar grouping approach with our study, Seki (18) reported the markedly higher cutoff of 1.9 x 10^-3^ mm^2^/sec (sensitivity 95.2%, specificity 50%, accuracy 77.1%). In lieu of classifying thymomas as low- and high-risk based on the WHO classification as we did, some foregoing studies (2, 19) divided them according to the Masaoka-Koga staging system into non-invasive (fully encapsulated, Masaoka-Koga stage I) and invasive (Masaoka-Koga stage II–IV). In particular, Gümüştaş had two non-invasive thymomas in the “benign” group and no invasive thymomas in the “malignant” group; the ADC cutoff distinguishing the “benign” and “malignant” groups was 1.39 x 10^-3^ mm^2^/sec (19). Nasr had four non-invasive thymomas in the “benign” group and five invasive thymomas in the “malignant” group; the ADC cutoff distinguishing the “benign” and “malignant” groups was 1.15 x 10^-3^ mm^2^/sec (2). Though the grouping method may be different, one similarity is shared across studies: TETs are heterogenous in biological behavior, prognosis, and treatment planning, so that they should not be included in one sole group.

**Figure 10:**
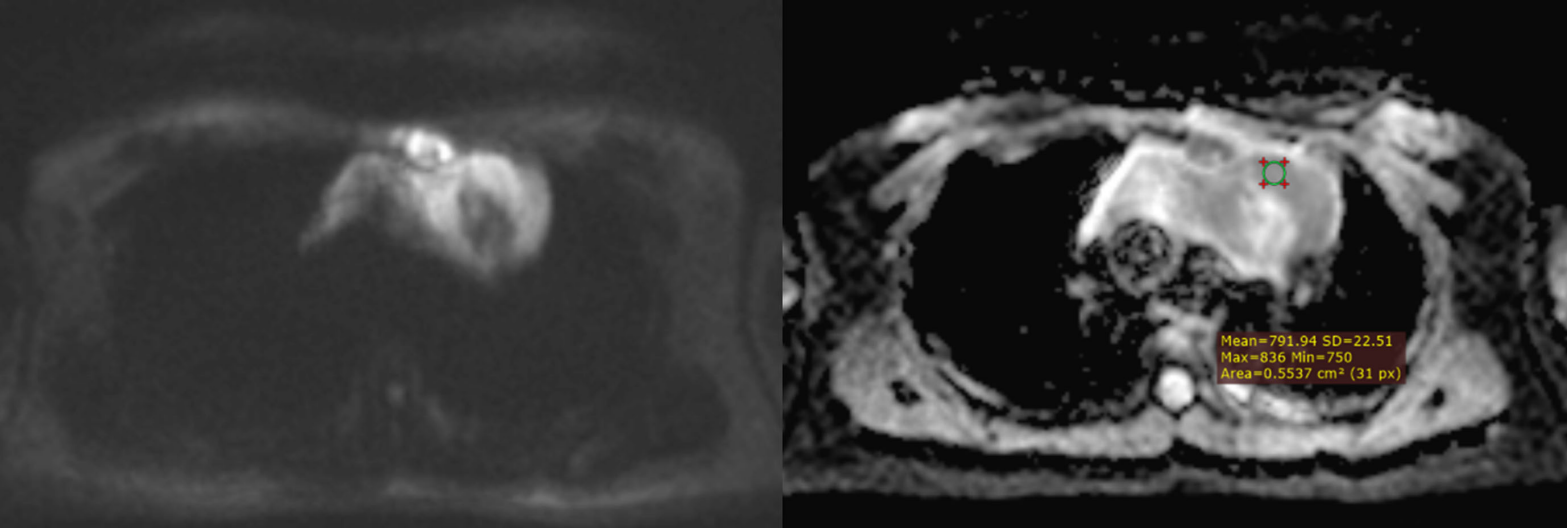
Mediastinal diffuse large B-cell lymphoma. A mediastinal diffuse large B-cell lymphoma having areas of restricted diffusion, which were hyperintense on DWI (b = 2000 sec/mm2) and correspondingly hypointense on ADC map. The mean of 3 ADC values obtained by using the hot-spot-ROI-based approach was 0.75 x 10-3 mm2/sec.

Zhang et al. reported that the hot-spot-ROI-based mean ADC of lymphoma was significantly lower than that of thymic carcinoma (20). In our study, the hot-spot-ROI-based mean ADC of lymphoma was significantly lower than that of other malignant mediastinal tumors (**Figures 9**, **10**). This may be explained by the characteristic histopathological features of lymphoma, which are hypercellularity and enlarged nuclei. Using the cutoff of 0.91 x 10^-3^ mm^2^/sec, the differentiation between lymphoma and other malignant tumors was achieved with a sensitivity of 100%, a specificity of 60.5% and an accuracy of 66%. Our cutoff is higher than the cutoff reported by Zhang et al, which was 0.73 x 10^-3^ mm^2^/sec. The variation in ADC cutoffs is likely attributed to the discrepancy in sample populations, ROI placement methods, and DWI acquisition parameters. The main difference between the hot-spot-ROI methods employed in the two studies is that we placed ROIs at the assumed most hypointense areas on the ADC map regardless of the lesion’s diameter, while Zhang placed ROIs on a single slice at which the tumors appeared largest (20). Additionally, their selection of b-values (0 and 1000 sec/mm^2^) was also different from ours. Our results are discordant with Gümüştaş, Razek, and Sabri; they all reported that there was no significant difference in the mean ADC value between lymphoma and other malignant tumors (19, 21, 22).

When whole-lesion histograms were used to derive ADC parameters, our study showed that the ADC_10_, ADC_90_, ADC_mean_, and ADC_median_ of group B were significantly lower than those of group A. Among the four parameters, the ADC_10_ demonstrated a mildly better performance; the optimal distinction was obtained when using the ADC_10_ cutoff of 0.85 x 10^-3^ mm^2^/sec (sensitivity 75.7%, specificity 72.2%, accuracy 74.6%). This is in agreement with the result of Kong et al, who reported that ADC_10_ was superior to mean, median, or 90th percentile ADC in differentiating tumors (20). In agreement with our result, Kong et al. demonstrated that ADC_10_ showed the best performance for discriminating low-risk thymoma from high-risk thymoma and thymic carcinoma (20). The superior performance of the low percentile ADCs obtained by the histogram approach was also demonstrated in characterizing tumors of other organs. Huang et al. found that the ADC_10_ was helpful in WHO grading of ependymoma (23). Donati et al. showed that ADC_10_ correlated with the Gleason score better than the other ADC parameters did, suggesting that the 10th percentile ADC may be the best for distinguishing low- from intermediate- or high-grade prostate cancer (24). In cases of tumors having heterogeneous morphology, focal areas with high cellularity were represented to a greater extent by the low percentile ADCs than the mean or median ADCs, resulting in the better differentiation performance of the ADC_10_ (25).

The ADCs obtained by the two approaches reflected the lesions’ cellularity and had satisfactory performance in distinguishing lesion groups. Yet, we noticed the discrepancies in the absolute ADC values of distinct pathologies and the absolute ADC cutoffs reported among studies (19, 21). The differences in absolute values can be explained by the dissimilarities in DWI acquisition parameters and how ROIs were placed on the ADC map. Priola et al. claimed that DWI acquisition parameters, particularly b-value and echo time (TE), could affect the reproducibility of ADC (16). Also, Iima stated that the ADC value decreased when high b-values were used (26). Using an ultra-high b-value of 2000 sec/mm^2^, the ADC values of specific lesion types as well as the ADC cutoffs to differentiate groups in our study were lower compared to those reported in the previous works (19, 21). Another reason for the ADC values in our study being lower is our hot-spot-ROI approach: the ROIs were placed on the most hypointense regions on the ADC map, which were correspondingly hyperintense on the trace image. We believe this approach is able to represent lesions (thanks to choosing the most hypercellular area) and is time-saving, so that it could be applied to everyday practice. To improve the reproducibility of ADC, standardization of DWI acquisition parameters and ROI placement methods should be implemented (16). By the time the consensus on chest DWI acquisition and interpretation is published, ADC cutoffs reported in research should be evaluated along with DWI acquisition parameters and how ROIs were placed on the ADC map. Mathematically, ADC calculation is independent of the strength of the magnetic field (27). In the present study, the images were acquired using the 3 Tesla field strength, which is believed to produce better spatial resolution in trace images and enable a shorter acquisition time compared to the 1.5 Tesla. The thorax is famous for its complex motions, so the short scan time achieved by the high field strength helps improve the image quality.

Our study had some limitations. First, the sample size was small; in particular, the quantity of benign lesions was modest compared to that of malignant cases. Second, the ADC values were extracted from the ADC maps by a single radiologist, so we could not investigate the inter-reader agreement. Third, the ADC values of each pathology and ADC discriminatory cutoffs are heavily influenced by DWI acquisition parameters and ROI placement methods, leading to the limitation of adopting them to clinical practice across different institutions. Fourth, there was a wide spectrum of histopathology in our study. Further studies with a large sample size focusing on a specific pathology are recommended.

## Conclusion

Diffusion-weighted imaging with ADC measurements is helpful in differentiating between malignant and benign lesions of the anterior mediastinum. Compared to the ADC parameters derived from whole-tumor histogram analysis, the mean ADC obtained by the hot-spot-ROI approach performed slightly better in distinguishing the lesion groups.

## Data availability statement

The original contributions presented in the study are included in the article/supplementary material. Further inquiries can be directed to the corresponding author.

## Ethics statement

The studies involving human participants were reviewed and approved by Institutional Review Board (IRB) of University of Medicine and Pharmacy at Ho Chi Minh City, Ho Chi Minh City 700000, Vietnam. Number approval: 739/HDDD-DHYD (Approval date: 22 October, 2020). The ethics committee waived the requirement of written informed consent for participation.

## Author contributions

Conceptualization, TT and VD; Data curation, TT and NN; Formal analysis, TT, PC, and LM; Investigation, TT, NT, TV, and PC; Methodology, TT; Project administration, TT and LM; Software, VD, PC, and LM; Supervision, VD and LM; Validation, LM; Writing – original draft, TT, NT, TV, VD, NN, PC, LN, and LM; Writing – review and editing, NN, LN, and LM. All authors contributed to the article and approved the submitted version.

## Conflict of interest

The authors declare that the research was conducted in the absence of any commercial or financial relationships that could be construed as a potential conflict of interest.

## Publisher’s note

All claims expressed in this article are solely those of the authors and do not necessarily represent those of their affiliated organizations, or those of the publisher, the editors and the reviewers. Any product that may be evaluated in this article, or claim that may be made by its manufacturer, is not guaranteed or endorsed by the publisher.
